# Physical, nutritional, and sensory quality of rice‐based biscuits fortified with safou (*Dacryodes edulis*) fruit powder

**DOI:** 10.1002/fsn3.1622

**Published:** 2020-05-16

**Authors:** Eliane‐Flore Eyenga, Erasmus Nchuaji Tang, Mercy Bih Loh Achu, Renaud Boulanger, Wilfred F. Mbacham, Sali Atanga Ndindeng

**Affiliations:** ^1^ Laboratory of Food Science and Technology Institute of Agricultural Research for Development (IRAD) Yaoundé Cameroon; ^2^ Faculty of Science University of Yaoundé‐1 Yaoundé Cameroon; ^3^ CIRAD UMR QUALISUD Montpellier cedex 5 France; ^4^ Africa Rice Center (AfricaRice) M’be Research Station Bouake Côte d’Ivoire

**Keywords:** fine broken rice, fortified biscuits, postharvest loss, quality, sour and nonsour safou

## Abstract

The reduction of postharvest losses in rice and safou is imperative to increase productivity in their respective value chains. In this study, fine broken rice grains were used to produce rice flour and subsequently rice‐based biscuits. The biscuits were further fortified with safou powder, and the physical, nutritional, and sensory quality and stability during storage of the different types of biscuits were analyzed using standard methods. Fine or nonsandy biscuits had peak particle size of 500 µm, while medium (slightly sandy) and large (sandy) biscuits had peak particle sizes of 1,000 µm and 1,400 µm, respectively. The hardness varied from 5.7 ± 2.3 N for biscuits with large particles to 16.1 ± 4.4 N for biscuits with fine particles. Fortification of biscuits with sour safou increased the protein and amino acid content of the biscuits. Tryptophan was absent in both safou and the biscuits produced. There was an increase in phosphorus, potassium, calcium, magnesium, copper, iron, manganese, and aluminum following fortification with safou. Nonsandy biscuits dissolved faster in the mouth (melt) during consumption than the other biscuits although most of the biscuits were perceived to be low in melting and buttery. Nonsandy biscuits were rated as “very good,” while slightly sandy and sandy were rated as “good.” Safou rice‐based biscuits were perceived as “very good,” while simple rice biscuits were perceived as “good.” Fortification of rice biscuits with safou increased the protein, essential amino acid, and mineral contents of the biscuits with very appreciable taste. These biscuits can be used to help fight protein, iron, and zinc malnutrition and in mitigating postharvest losses of underutilized broken rice and safou especially sour safou.

## Graphical abstract

In this paper, we show that fine broken rice can be used to produce biscuits which are highly acceptable by consumers. We further demonstrate that such biscuits can be fortified with protein, amino acids, and trace minerals such as iron and zinc by partially substituting butter with safou—an oleaginous fruit widely cultivated in West Africa. The use of fine broken rice and safou to produce biscuits will result in a win‐win scenario for both rice and safou value chains through postharvest loss reduction and value addition.

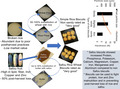

**Abbreviations**
IRRI: International Rice Research Institute
MDA: malondialdehyde
NSSRWB‐50: nonsour safou–rice–wheat biscuit
RB‐100: rice biscuit
RWB‐50: rice–wheat biscuit
SSRWB‐50: sour safou–rice–wheat biscuit
WB‐100: wheat biscuit

## 1 INTRODUCTION

Rice (*Oryza sativa L*.) is currently part of the staple foods in Cameroon with close to 896,000 tons consumed in 2018. Milled rice production in Cameroon in 2018 stood at 221,000 tons which is a 2.21% reduction compared to 2016 (International Rice Research Institute (IRRI), 2020). Poor pre‐ and postharvest practices have resulted in low productivity of rice, and this has led to the importation of rice to meet national demand. Locally produced rice contains a large proportion of broken grains (35%–63%) due to inadequate methods of harvesting, threshing, drying, and milling (Mapiemfu et al., 2017;Ndindeng, Manful, et al., 2015). This low quality also affects the price and choice of consumers, because they prefer rice of superior quality even if they pay a higher price (Akoa‐Etoa et al., 2016). Broken rice can be ground to produce flour with different particle sizes. This flour can be transformed into paste, porridge, doughnut, cake, pancake, and biscuits. Biscuit is the bakery product widely accepted and appreciated around the world. It is the most desirable snack for both young and adults, and it is also easy and cheap to produce (Akubor, 2003;Hooda & Jood, 2005). Biscuits are rich in carbohydrates, lipids, and calories but low in dietary fibers, minerals, and vitamins, which makes them not suitable for daily consumption (Serrem, De Kock, & Taylor, 2011). The nutritional value of biscuits can be improved through fortification and supplementation using a wide variety of proteins, lipids, and mineral sources (Induja, Pornoor, Maitrayee, & Vandana, 2012). Substituting wheat with rice flour from broken grains will generate diversified foods with unique qualities while adding value to the domestic rice value chain. In addition, unlike wheat, rice is a gluten‐free cereal that contains low levels of sodium and a high percentage of digestible carbohydrates (Rai, Kaur, & Chopra, 2018). This makes it desirable in the preparation of gluten free diets to reduced wheat allergies and celiac disease especially in infants (Arendt and Dal Bello, 2008).

The safou (*Dacryodes edulis* (G. Don H. J. Lam)) fruit is mostly found in forest areas of Central and West Africa and the Gulf of Guinea. Safou is mostly eaten in the roasted or boiled form, as a delicacy alone or in combination with roasted or boiled maize, cassava or plantain. The safou pulp is rich in lipids, and its proportions vary from 33% to 65%. Safou lipids contain a high level of essential fatty acids (Dzondo‐Gadet, Nzikou, Etoumongo, Linder, & Desobry, 2005). Safou is also rich in potassium, phosphorus, calcium, magnesium, and sodium (Ene‐Obong, Igile, Ekpo, Egbung, & Agbo, 2019). Depending on the taste, there are two types of safou: the nonsour and the sour safou (Ndindeng, Bella‐Manga, Kengue, Talle & Lewis, 2008). The nonsour safou is highly appreciated by consumers and mostly consumed in the fresh form. However, the sour safou is mostly abandoned and constitutes a major postharvest loss (Ndindeng, Talle, Bigoga, Kengue, Boffa J.‐M., 2012). In Cameroon, safou records closed to 50% postharvest loss (Ndindeng, Kengue, Mbacham, Titanji, and Bella‐Manga (2007)) because it rapidly degrades during storage for 2–3 days at room conditions (T = 25°C, RH = 75%). Technologies to produce dried safou have been developed, but the consumption of dried fruits is not customary in Cameroon and other Sub‐Sahara African (SSA) countries. Dried safou pulp can be ground and used as an ingredient in the production of biscuits but the acceptability of such biscuits and expected nutritional benefits are unknown. Fortification of rice‐based biscuits with safou is important in order to reduce the high postharvest loss of safou (Ndindeng et al., 2007;Ndindeng, Talle, & J., Kengue, J., Boffa J.‐M., 2012) and to add value to broken rice grains. This will lead to a win‐win scenario as postharvest loss for both safou and rice will be reduced, and acceptability of the end products is expected to be high. This study is therefore aimed at formulating rice‐based biscuits fortified with safou and assessing the nutrient composition and the sensory quality of the biscuits.

## 2 MATERIALS AND METHODS

### 2.1 Production of rice‐based biscuits fortified with safou

#### 2.1.1 Collection of safou and rice samples

Sour and nonsour safou clones that had previously been characterized for taste (Ndindeng et al., 2008) were harvested at the Institute of Agricultural Research (IRAD) experimental farm at Nkolbisson, Yaoundé‐Cameroon. Fine broken rice of TOX‐3,145‐TOC‐38–2–3 variety, which is predominantly grown by farmers in the Ndop Rice Development Hub (rice hub), Northwest and Western Regions of Cameroon (AfricaRice, 2018), was purchased from commercial millers.

#### 2.1.2 Production of safou powder

The safou fruits were washed with distilled water and then cut into two halves using a knife. The seeds and pericarp were removed. The fruits were sliced into 1.5 cm^2^ and dried in a Heraeus® D‐6450 Hanau drying oven for 24 hr at 50–60°C. The chips were removed and cooled to room temperature, crushed in a blender, packaged in plastic bags, and stored in a refrigerator at 4°C until use.

#### 2.1.3 Production of rice flour

Fine broken rice from the locality of Ndop, Northwest Region of Cameroon, was winnowed then washed and soaked in water for 8 h. The rice was removed from water and dried to moisture content of 20%. It was then crushed in a regular corn mill (dry milling) before completely drying the flour to 10%–12%. Rice flour was produced by sieving ground rice with sieves of different mesh sizes as previously described by Ndindeng, Mbassi, et al. (2015). Briefly, each sample was separated into three particle sizes (large = particle sizes ≥ 1 mm; medium = 0.301–0.99 mm; fine ≤ 0.300 mm) using sieves of mesh size 18 and 50. The sample was first sieved with size 18 and then with size 50. Particles remaining in sieve 18 were large, while those that remained in sieve 50 were of medium size, and those that passed through sieve 50 were the fine particles.

#### 2.1.4 Types of biscuits produced

**Figure 1:**
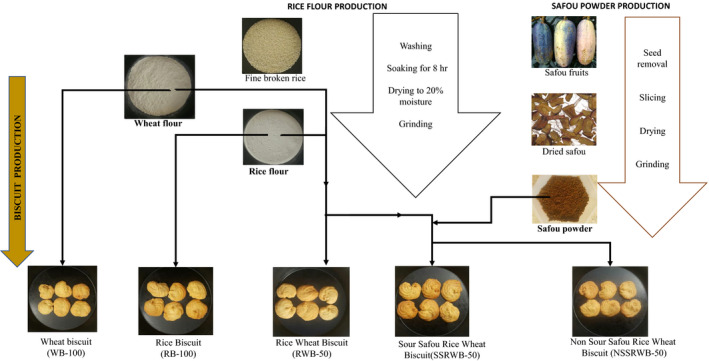
Schematic presentation of the rice flour, safou powder, and different biscuit production processes

Three types of biscuits were produced with rice flour of different particle sizes. Large, medium, and fine particles were used to produce sandy, slightly sandy, and nonsandy simple rice biscuits (RB100), respectively. Six different wheat, rice, and safou nonsandy biscuit combinations were produced using fine flour. These were wheat biscuit (WB‐100), rice biscuit (RB‐100), rice–wheat biscuit (RWB‐50), sour safou–rice–wheat (SSRWB‐50), and nonsour safou–rice–wheat (NSSRWB‐50) biscuits (Figure 1). The ingredients for each type of biscuit are shown in Table 1.

**Table 1:** Proportion of ingredients used to produce wheat, rice, and safou composite biscuits

Ingredients	Types of biscuits					
	Wheat (WB−100)	Rice (RB−100)	Rice safou (RSB−100)	Rice–wheat (RWB 50%)	Sour safou–rice–wheat (SSRWB−50)	Nonsour safou–rice–wheat (NSRWB−50)
Rice flour (g)	0	1,200	600	600	600	600
Wheat flour (g)	600	0	600	600	600	600
Butter (g)	600	600	480	600	480	480
Sugar (g)	400	400	400	400	400	400
Eggs (g)	400	400	400	400	400	400
Baking powder (g)	10	10	10	10	10	10
Salt (g)	4	4	4	4	4	4
Aroma (vanilla) (g)	20	20	20	20	20	20
Safou powder (g)	0	0	120	0	120	120

#### 2.1.5 Biscuit production process

The proportion of wheat, rice, safou, and butter for biscuit production was optimized through sensory evaluation using the Doehlert design in XLSTAT Premium version 2020.1.2 (Addinsoft, 2020). The equations for each response attribute, the response optimization parameters, and local solutions are shown in Supplementary 1. For biscuit production, butter was put in a clean bowl and creamed thoroughly with a stainless steel spatula to become soft and smooth. Powdered sugar was added and creamed to give a homogenous mixture. In recipes with safou, the safou was added to the butter–sugar mixture and properly mixed to obtain a smooth paste. Flour was mixed together with baking powder and salt. This flour–baking powder–salt mixture was added to the butter–sugar (safou) mixture and homogenized with the spatula to form a paste. Egg and liquid flavor were then added, and the mixture stirred to obtain the final biscuit dough. The dough was then put into the biscuit mold, and the desired biscuit shape formed on a tray lightly greased with butter. The filled trays were introduced into an oven (Panasonic MOV‐212) set at 160°C and baked for 20–25 min. The light brown baked biscuits obtained were removed and put in a large tray to cool to room temperature before packaging.

#### 2.1.6 Evaluation of Biscuits during storage

Wheat (WB‐100), rice (RB‐100), rice safou (RSB‐100), rice–wheat (RWB‐50), sour safou–rice–wheat (SSRWB‐50), and nonsour safou–rice–wheat (NSSRWB‐50) biscuits were produced, packaged in either aluminum or polypropylene bags and stored for 5 months at room conditions (T = 25°C, RH = 60%). Every month during the storage period, samples were withdrawn and evaluated for hardness, water content, malondialdehyde content, peroxide index, lightness, and aroma.

### 2.2 Analysis of biscuits

#### 2.2.1 Physical analysis of biscuits

The particle size distribution of rice biscuits was determined by sieving ground biscuits with sieves of different meshes from 1.4 µm to 250 µm (sieve vibration Analysette 3 Pro, Fritsch). The hardness of the biscuits produced was determined through the measurement of the penetration strength with the Texturemeter Stevens Instron3343 at room temperature. The experimental equipment was made up of a borer‐like cone, falling at 0.2 mm/s of speed until the biscuit was broken. The lightness of the biscuit was measured using a Colorimeter (Minolta Color reader CR‐10) and reported as L*value. This lightness was measured by placing the aperture of the equipment on the sample with white paper as the reference. The L* value was calculated automatically and expressed as a difference (Navneet & Shitij, 2011;Ndindeng, Manful, et al., 2015).

#### 2.2.2 Chemical analysis of safou powder, simple rice, and simple rice safou biscuits

These analyses were done to reveal the differences between sour and nonsour safou and biscuits produced with these safou. The water content was determined by the gravimetric method (Künsch et al., 1999). Lipid extraction was performed using AvantiSoxtec with petroleum ether as the solvent (Anon, 1990). Total nitrogen was determined by the Kjeldahl method using 6.25 as the nitrogen conversion factor to total protein. The contents in total amino acids were determined by analytical protocol for total amino acids using Biochrom 30 + analyzer (Biochrom Ltd, Cambridge, United Kingdom). Carbohydrate was determined by the Anthrone method (Leyva et al., 2008), and dietary fiber was determined according to Garbelotti, Marsiglia, and Torres (2003). The mineral content (P, K, Ca, Mg, Na, Cu, Fe, Mn, Zn, Al) was determined by atomic absorption spectrophotometry (Varian Vista, Victoria, Australia)**.** The energy values were calculated theoretically using the following conversion factors 4.0, 4.0, and 9.0 kcal/g for protein, carbohydrates, and fat, respectively, according to the method described by Paul and Southgate (1979). Lipid oxidation was determined through the measurement of peroxide and malondialdehyde. The peroxide index was determined in the oil extracts of biscuits obtained through cold extraction with hexane and methylene chloride as solvents, following the official method NFT 60–220 of the Association Française de Normalisation (1995), while malondialdehyde was determined spectrophotometrically as described by Mendes, Cardoso, and Pestana (2009).

#### 2.2.3 Sensory evaluation of biscuits

The sensory attributes of the biscuits evaluated were the intensity of aroma, basic taste (sweet, salty, acidic, bitter), texture (granular, melty, greasy, crumbly, crunchy), and overall quality. A trained panel of 12 (2 men and 10 women) used a six‐point rating scale (“0 = absent” to “5 = very high”) to evaluate the intensity of basic taste, aroma, and texture. A six‐point scale was also used to evaluate the overall quality of the biscuits from “0 = very bad” to “5 = excellent.”

### 2.3 Statistical analysis

Radar charts were generated in Excel 2018 software (Office 365, Microsoft Corp.) from the results of sensory evaluation to depict differences in attributes between the formulated biscuits, based on particle size and safou fortification. Analysis of variance (ANOVA) with Fisher's pairwise comparison test was performed to determine the differences between the nutrient composition of dry safou powder and the biscuits. Analysis of covariance was used to study the effect of storage time, type of biscuits and package on hardness, malondialdehyde, moisture, peroxide index, and lightness during storage. Differences between categories of biscuits and packages were analyzed using the Fisher least significant difference. Analysis was done with XLSTAT Premium version 2020.1.2 (Addinsoft, 2020) with significant level of 5%.

## 3 RESULTS AND DISCUSSIONS

### 3.1 Physical analysis of biscuits in relation to the particle size

The particle size distribution in each category of biscuits was heterogeneous (Table 2). Fine or nonsandy biscuits had peak particle size of 500 µm, medium (slightly sandy) had peak particle size of 1,000 µm, while large (sandy) biscuits had peak particle size 1,400 µm (X^2^ = 89.38; *df* = 8, *p* < .0001). Particle size distribution has been shown to depend on the type of milling (wet or dry) and the machine used for grinding **(**Nishita & Bean, 1982 **).**

**Table 2:** Particle size distribution of sandy, slightly sandy, and nonsandy biscuits (RB‐100)

Type of rice biscuit	Sieve mesh size				
	1,400 μm	1,000 μm	500 μm	250 μm	125 μm
Nonsandy (fine) (g)	0	20.6	66.2	13.3	0
Slightly sandy (g)	37.7	49.1	13.3	0	0
Sandy (g)	60.7	34.2	4.0	0	0
	Chi‐square = 89,38; DF = 8; *p* < .0001				

The textural properties of the three types of biscuits are shown in Table 3. Hardness and cohesiveness were significantly different (*p* < .05) from nonsandy to sandy biscuits. The hardness was higher for nonsandy biscuits (16. 1 ± 4.4) and lower for sandy biscuits (5.7 ± 2.3). The cohesiveness varied from 7.18 ± 2.68 for nonsandy biscuits to 1.17 ± 0.70 for sandy biscuits. These observations demonstrate that the smaller the particles, the greater the cohesion of the particles. Lai (2001) also showed that cohesion could be influenced by the size of the granules, the characteristics of the starch molecules, and the thermal process involved in starch gelatinization.

**Table 3:** Textural properties of sandy, slightly sandy, and nonsandy biscuits (RB‐100)

Types of biscuit	Parameters	
	Hardness (*N*)	Cohesiveness
Nonsandy	16.1 ± 4.4^a^*	7.18 ± 0.61^a^
Slightly sandy	8.89 ± 2.3^b^	2.19 ± 6.68^b^
Sandy	5.7 ± 2.3^c^	1.17 ± 0.70^c^

* Indicates that means in the same rows with different letter superscripts are significantly different at *p* < .05.

### 3.2 Chemical analysis of sour and nonsour safou powder, rice–wheat biscuit, and sour safou and nonsour safou–rice–wheat biscuit

The proximate composition of sour safou, nonsour safou, simple rice–wheat biscuits, and safou‐fortified biscuits are shown in Table 4. The water content of the sour safou was higher (4.77 ± 0.18%) than that of nonsour safou (3.6 ± 0.23%). The water content of simple rice biscuits was higher than that for rice safou biscuits. This shows that the water content decreases with the addition of safou to rice–wheat biscuits especially nonsour safou. The low water content may increase the quality and stability of biscuits fortified with safou powder as previously reported for other foods (Onwuka, 2005). The lipid content of the nonsour safou was significantly higher (65.72 ± 0.91%) than that of sour safou (60.56± 0.44%), and this is likely related to the great variability reported in this tree crop (Leakey et al., 2005). However, the substitution of butter with safou powder did not significantly change the lipid content of those biscuits. The protein content of the sour and nonsour safou was 8.2 ± 0.01 and 9.03 ± 0.04%, respectively. However, nonsour safou–rice–wheat biscuits recorded a 52% increase in protein. Mbofung, Silou, and Mouragadja (2002) reported a 39% increase in protein after partially substituting safou for margarine in the production of biscuits. In that study, the authors did not indicate the type of safou (sour or nonsour) used in the substitution. The carbohydrate content of sour safou (23.53 ± 0.81%) was significantly higher (*p* < .05) than that of nonsour safou (14.07 ± 1.24). The safou biscuits had lower carbohydrate contents (53%–60.9%) than that of simple rice–wheat biscuit (62.87 ± 1.55%). However, the difference between the carbohydrate level of the sour safou rice biscuit and the simple rice–wheat biscuit was not significant. The crude fiber content of nonsour safou (7.6 ± 0.18%) was higher (*p* < .05) than that of sour safou (1.9 ± 0.19%). The crude fiber content of simple biscuits was slightly higher than those of safou‐fortified biscuits, but with no significant difference from that of nonsour safou biscuits. These observations are like those made by Noor Aziah, Mohamad Noor, and Ho (2012) who reported a decrease in the fiber content of biscuits when enriched with legume (mung bean and chickpea) flour. The energy value of nonsour safou was similar to that of sour safou (683 and 681 Kcal/g). The energy value of sour safou–rice–wheat biscuit (504.96 kcal/g) and nonsour safou–rice–wheat biscuit (497.13 kcal/g) was similar to that of simple rice–wheat biscuit (507.6 kcal/g).

**Table 4:** Proximate composition of safou powder and rice–wheat biscuits fortified or not fortified with safou

Material		Parameters					
		Water content (%)	Fat (%)	Protein (%)	Carbohydrate (%)	Crude fiber (%)	Energy (kcal/g)
Types of Safou	Sour	4.77 ± 0.18^b^	61,56 ± 0.44^a^	8.23 ± 0.01^a^	23.53 ± 0.81^b^	1.9 ± 0.19^a^	681.11 ± 0.7^a^
	Nonsour	3.6 ± 0.23^a^	65.72 ± 0.91^b^	9.03 ± 0.04^b^	14.07 ± 1.24^a^	7.6 ± 0.18^b^	683.37 ± 4.21^a^
Types of biscuit	RWB−50	4.80 ± 0.01^c^	25.41 ± 0.86^a^	6.71 ± 0.63^a^	62.87 ± 1.55^b^	2.77 ± 0.09^b^	507.6 ± 4.07^a^
	SSRWB−50	3.21 ± 0.00^b^	25.37 ± 0.00^a^	8.25 ± 0.00^b^	60.90 ± 0.09^b^	2.26 ± 0.09^a^	504.96 ± 0.43^a^
	NSRWB−50	2.9 ± 0.06^a^	25.25 ± 0.51^a^	13.71 ± 0.04^c^	53.75 ± 0.57^a^	2.47 ± 0.12^ab^	497.13 ± 2.11^a^

Note

RWB‐50 = rice–wheat; SSRWB‐50 = sour safou–rice–wheat; NSRWB‐50 = nonsour safou–rice–wheat.

* Indicates that means in the same column with different letter superscripts are significantly different at *p* < .05.

The essential and nonessential amino acid composition of different types of safou and biscuits is shown in Table 5. Leucine was the most abundant essential amino acid in safou, while aspartate was the most abundant nonessential amino acid. The essential amino acids—phenylalanine, isoleucine, histidine, tyrosine, and lysine—contents were higher in nonsour than sour safou. However, after addition of safou to rice–wheat biscuits, differences were observed only for histidine (higher content in NSRWB‐50) and lysine (lower content in NSRWB‐50). The nonessential amino acids—aspartate, glycine, alanine, gamma‐amino butyrate (GABA), proline, and arginine—contents were also higher in nonsour than sour safou. The addition of safou to rice–wheat biscuit combinations increased only the levels of aspartate and glycine. These results suggest that the differences in the taste of safou accessions were due to observed differences in its chemical composition, especially water, lipid, and amino acid contents. Tryptophan was absent in both safou and biscuits produced. Essential and nonessential amino acids are the building blocks of nitrogen containing molecules in living organisms. These amino acids are critical for cell repair, tissue synthesis, cellular regeneration, and calorie provision in periods of starvation (Razak, Begum,Viswanath, & Rajagopal, 2017).

**Table 5:** Amino acid contents of different types of safou and rice–wheat composite biscuits fortified with different types of safou

Material	Essential amino acids (g/100 g)								
	Phenylalanine	Valine	Threonine	Isoleucine	Methionine	Histidine	Leucine	Lysine	Tyrosine
Sour safou	0.65 ± 0.00^a^*	0.75 ± 0.05^a^	0.65 ± 0.14^a^	0.69 ± 0.03^a^	0.31 ± 0.01^a^	0.50 ± 0.02^a^	1.19 ± 0.01^a^	0.79 ± 0.02^a^	0.90 ± 0.02^a^
Non sour safou	0.83 ± 0.07^b^	0.93 ± 0.13^a^	0.72 ± 0.01^a^	0.91 ± 0.12^b^	0.36 ± 0.07^a^	0.69 ± 0.01^b^	1.62 ± 0.11^a^	0.96 ± 0.08^b^	1.21 ± 0.06^b^
RWB‐50	0.43 ± 0.00^a^	0.41 ± 0.14^a^	0.31 ± 0.01^a^	0.33 ± 0.01^a^	0.25 ± 0.01^a^	0.26 ± 0.00^a^	0.69 ± 0.00^a^	0.31 ± 0.00^b^	0.32 ± 0.01^a^
SSRWB‐50	0.45 ± 0.02^a^	0.43 ± 0.04^a^	0.33 ± 0.03^a^	0.35 ± 0.04^a^	0.29 ± 0.02^a^	0.29 ± 0.02^ab^	0.74 ± 0.03^a^	0.32 ± 0.01^b^	0.38 ± 0.01^a^
NSRWB‐50	0.42 ± 0.05^a^	0.41 ± 0.07^a^	0.29 ± 0.04^a^	0.27 ± 0.02^a^	0.24 ± 0.02^a^	0.32 ± 0.01^b^	0.69 ± 0.04^a^	0.26 ± 0.01^a^	0.35 ± 0.03^a^

Types of biscuits	Non‐essential amino acids (g/100 g)								
	Aspartate	Serine	Glutamate	Glycine	Alanine	Gamma amino Butyrate	Proline	Arginine	Cystine
Sour safou	2.00 ± 0.05^a^*	0.83 ± 0.14^a^	1.71 ± 0.11^a^	0.77 ± 0.07^a^	0.83 ± 0.07^a^	0.05 ± 0.00^a^	1.01 ± 0.04^a^	0.77 ± 0.05^a^	0.19 ± 0.03^a^
Non sour safou	2.78 ± 0.04^b^	1.01 ± 0.02^a^	2.44 ± 0.26^a^	1.07 ± 0.04^b^	1.19 ± 0.05^b^	0.11 ± 0.01^b^	1.25 ± 0.04^b^	1.07 ± 0.05^b^	0.19 ± 0.00^a^
RWB‐50	0.65 ± 0.01^a^	0.54 ± 0.01^ab^	2.3 ± 0.02^a^	0.36 ± 0.00^a^	0.43 ± 0.00^a^	0.00 ± 0.00^a^	0.68 ± 0.14^a^	0.45 ± 0.01^a^	0.17 ± 0.02^a^
SSRWB‐50	0.79 ± 0.01^b^	0.61 ± 0.00^b^	2.33 ± 0.06^a^	0.4 ± 0.00^b^	0.5 ± 0.00^b^	0.00 ± 0.00^a^	0.75 ± 0.00^a^	0.51 ± 0.01^a^	0.19 ± 0.01^a^
NSRWB‐50	0.79 ± 0.02^b^	0.51 ± 0.00^a^	2.15 ± 0.23^a^	0.41 ± 0.00^b^	0.43 ± 0.01^a^	0.00 ± 0.00^a^	0.67 ± 0.07^a^	0.46 ± 0.04^a^	0.18 ± 0.01^a^

Note

RWB‐50 = Rice–wheat; SSRWB‐50 = sour safou–rice–wheat; NSRWB‐50 = non‐sour safou–rice–wheat.

* Indicates that means in the same rows with different letters are significantly different at the *p* < .05.

The mineral composition of sour safou, nonsour safou, safou‐fortified, and simple biscuits are presented in Table 6. Potassium is the most abundant mineral in safou (0.819 g/100 g) followed by magnesium, phosphorus, and calcium with sodium being the least. Iron was the most abundant trace mineral in safou (22.6 ppm) followed by copper, manganese, aluminum, and zinc. In rice biscuits, potassium was still the most abundant mineral (0.166 g/100 g) followed by phosphorus. Although trace minerals iron and zinc are higher in biscuits than in safou, there was a higher quantity of aluminum in biscuits (214.8 ppm) than in safou. The quantity of aluminum recorded in biscuits produced in this study was similar or lower to that recorded for other biscuits (Diet Grail, 2020). Aluminum in the food supply comes from natural sources including water, food additives, and contamination by aluminum utensils and containers. The daily intake of aluminum varies greatly from 0 to 0.095 g, and the real question is not the amount of aluminum in foods but the availability of the aluminum in foods and the sensitivity of some population groups to aluminum (Greger, 2007). Potassium is a mineral and an electrolyte that the body needs to work properly. Potassium is a very important mineral for the human body as it is needed to build proteins, break down and use carbohydrates, build muscle, maintain normal body growth, control the electrical activity of the heart, and control the acid–base balance. The required daily intake (RDI) is age depended—generally 0.4–0.8 g/day for infants, 2–2.3 g/day for adolescent, and 2.6–3.4 g/day for adult (United State Department of Agriculture (USDA), 2020). Iron is a mineral found in every cell of the body. Iron is considered an essential mineral because it is needed to make hemoglobin, a part of the blood cell. The human body needs iron to make the oxygen‐carrying proteins hemoglobin and myoglobin. Hemoglobin is found in red blood cells, and myoglobin is found in muscles. The RDI is age‐ and sex‐dependent—except for infants younger than 6 months whose recommended intake is 0.00027 g/day, the recommended intake is generally between 0.007 and 0.018g/day (USDA, 2020). Zinc is found in cells throughout the body. It is needed for the body's defensive (immune) system to properly work. It plays a role in cell division, cell growth, wound healing, and the breakdown of carbohydrates. Zinc is also needed for the senses of smell and taste. During pregnancy, infancy, and childhood, the body needs zinc to grow and develop properly (Jarosz, Olbert, Wyszogrodzka, Młyniec, & Librowski, 2017). Zinc also enhances the action of insulin and is a potent inhibitor of coronavirus and arterivirus replication (te Velthuis et al., 2010). The RDI is also age‐ and sex‐dependent—generally, 0.002–0.008 g/day for infant and children and 0.011–0.013 g/day for adolescent and adults (USDA, 2020). In cereals such as rice and wheat, zinc bioavailability is depressed by phytic acid. Thus, the biscuits produced can be a good source for potassium, phosphorus, iron, and Zn for different age and sex groups.

**Table 6:** Variation in mineral content of safou and rice–wheat composite biscuits fortified and not fortified with safou.

Materials		Mineral content									
		P (g/100g)	K (g/100g)	Ca (g/100g)	Mg (g/100g)	Na (g/100g)	Cu (ppm)	Fe (ppm)	Mn (ppm)	Zn (ppm)	Al (ppm)
Types of safou	Sour safou	0.128	0.941	0.165	0.133	0.003	15.1	27.6	10.5	8.9	13.1
	Nonsour safou	0.123	0.697	0.085	0.137	0.003	20.4	17.6	10.3	10.9	6.9
	Mean	0.126	0.819	0.125	0.135	0.003	17.8	22.6	10.4	9.9	10.0
	*SD*	0.003	0.122	0.040	0.002	0.000	2.7	5.0	0.1	1.0	3.1
Types of biscuit	RWB−50	0.115	0.135	0.029	0.024	0.167	1.4	31.7	4.5	35.3	215.9
	SSRWB−50	0.132	0.186	0.041	0.033	0.164	2.5	35.9	5.1	33.8	223.4
	NSRWB−50	0.129	0.176	0.034	0.032	0.161	2.6	36.7	5.1	34.2	205.3
	Mean	0.125	0.166	0.035	0.030	0.164	2.2	34.7	4.9	34.4	214.8
	*SD*	0.007	0.022	0.005	0.004	0.002	0.5	2.1	0.2	0.6	7.4

Note

RWB 50% = rice–wheat; SSRWB‐50 = sour safou–rice–wheat; NSRWB‐50 = nonsour safou–rice–wheat.

Copper and manganese are also important trace minerals, and their levels are much higher in safou than rice–wheat biscuits produced. Copper is the cofactor of cuproenzymes essential for critical biochemical reactions in living cells and is required for red and white blood cell maturity and iron transport. Its low levels in the body are associated with Cu‐deficiency anemia that leads to impaired iron mobilization (Sandstead, 1995). Manganese is a cofactor in several metalloenzymes like superoxide dismutase and phosphoenol‐pyruvate carboxykinase involved in protecting the cell against reactive oxygen species and carbohydrate metabolism respectively (Li & Yang., 2018). In animals, manganese deficiency is responsible for impaired growth, skeletal abnormalities, ataxia, and defects in carbohydrate and lipid metabolism (Horning, Caito, Tipps, Bowman, & Aschner, 2015). The RDI of manganese for infants, children, and adolescents and adults is 0.0003–0.001 g/day, 0.001–0.003 g/day, and 0.002–0.005 g/day, respectively (USDA, 2020).

### 3.3 Sensory evaluation

**Figure 2:**
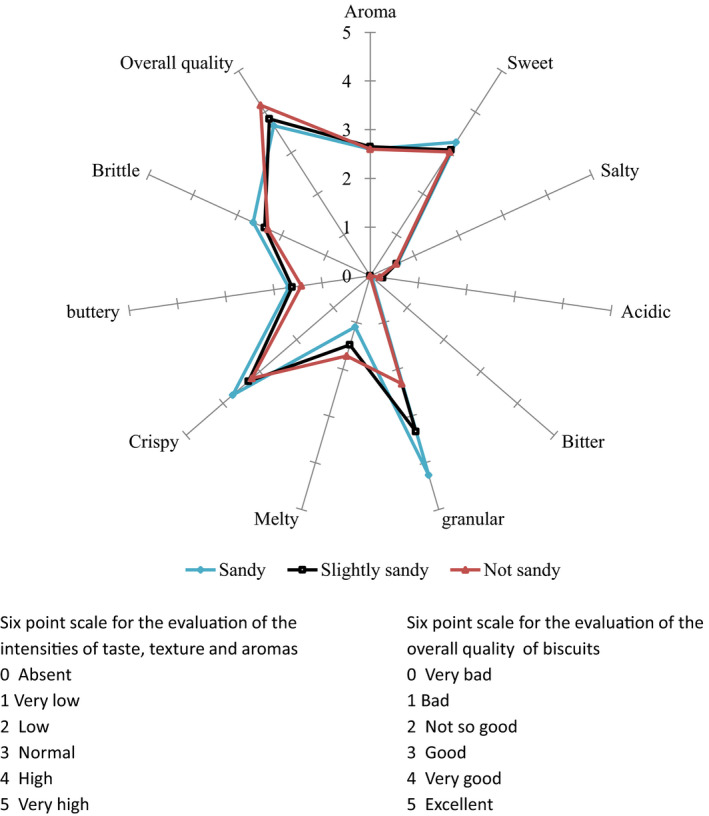
Sensory properties and acceptability of rice biscuits (RB‐100) depending on the size of the particles

The acceptability of rice biscuits (RB‐100) based on particle size distribution is shown in Figure 2. The point scores for brittle (2.64), buttery (1.69), and crispy (3.74) attributes were highly expressed in the sandy or large particle size biscuits than in the slightly sandy (2.38, 1.62, 3.31) and fine particle size or not sandy (2.30, 1.43, 3.21) biscuits, respectively. The overall quality score showed that the nonsandy were highly rated (4.17) followed by the slightly sandy (3.83) and lastly by the sandy (3.67) biscuit on a scale of 5. This implied that the acceptability of RB‐100 increased when the flour particle size got smaller (*p* < .05). Flour particle size is an important factor affecting protein quality, damaged starch content, and quality of bakery products **(**Barak, Mudgil, & Khatkar, 2014;Zohoun et al., 2018).

**Figure 3:**
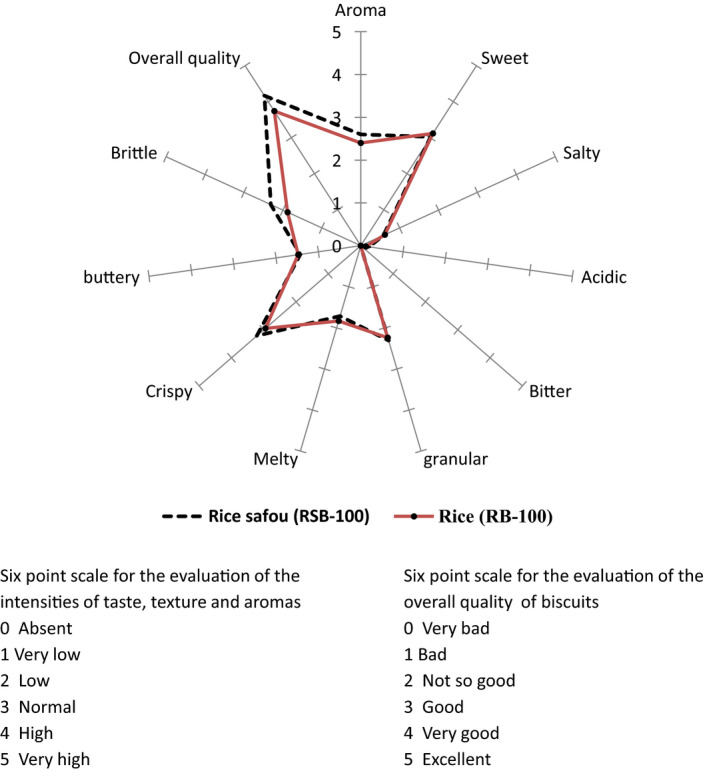
Sensory properties and acceptability of rice safou (RSB‐100) and rice (RB‐100) biscuits produced with fine particles

The sensory evaluation results of the safou fortified and unfortified biscuits produced using the fine particle size (not sandy) composite rice–wheat flour is shown in Figure 3. Sweetness recorded the same score in the safou fortified and simple rice biscuits (3.2 points). Acidity was not felt, recording a score of zero in both the unfortified and fortified biscuits. The aroma of the safou fortified biscuits (2.60) scored higher than that of unfortified samples (2.4). Safou fortified biscuits were more brittle (2.31 points) than simple rice biscuit (1.88). Therefore, the incorporation of safou powder into the biscuit formulation reduced the bonding forces between the different constitutive elements, making the biscuits fragile. The substitution of butter with 30% safou powder affected the crispiness of the biscuits, which is an important kinesthetic characteristic. The results showed that supplementation with safou increased crispiness. Crispiness is perceived when food is chewed between molars and is usually expressed in terms of hardness and fracturability (Mihiranie, Jayasundera, & Perera, 2017). Overall acceptability combines all the attributes covering individual judgment by the panelists. For overall acceptability, safou fortified biscuits scored higher (4.16) than the simple rice biscuits (3.74), an indication that the incorporation of safou into biscuit formulation improves its quality (*p* < .05). Safou (sour or nonsour) fortified biscuits were perceived as “very good,” while unfortified biscuits were perceived as “good.” This was probably due to the presence of the safou powder which enhanced the flavor with the characteristic safou aroma. Results from earlier studies in food fortification hold that consumers favorably embrace the incorporation of new and innovative nutrient rich supplements into biscuits. These supplements among others include protein‐rich palm weevil larvae (*Rhynchophorus phoenicis* Fabricius), orange‐fleshed sweet potato (Ayensu, Lutterodt, Annan, Edusei, & Loh, 2019), bambara nut, and aerial yam (Uchenna & Omolayo, 2017).

### 3.4 Stability of the biscuits during storage

Biscuit shelf‐life indicators as affected by duration of storage, type of biscuit, and packaging material are presented in Table 7. The NSSRWB‐50 biscuit and the polypropylene package were used as the references. The hardness of the biscuits did not change during storage and the packaging material did not affect the hardness. The malondialdehyde (MDA) value increased with the duration of storage and was significantly lower in WB‐100, RWB‐50, and SSWRB‐50 compared to NSRWB‐50 and RB‐100 (*p* < .05). MDA was also lower in biscuits stored in aluminum packages compared to those stored in polypropylene. The increase in MDA with duration of storage observed in this study confirms an earlier observation by Fotouo‐M, Vorster, du Toit, and Robbertse (2018) who showed higher MDA in *Moringa oleifera* oilseeds packaged in paper bags than in those packaged in aluminum bags when both were stored for 24 month at 20°C. The moisture content increased in biscuits during storage as observed with the storage of rice and other cereals (Tang et al., 2019). WB‐100 recorded higher moisture content, while RWB‐50 and SSRWB‐50 recorded lower moisture content compared to RB‐100 and NSRWB‐50 (*p* < .05). Biscuits stored in aluminum packages recorded lower moisture content compared to those stored in polypropylene bags (*p* < .05). The increase in moisture content with the duration of storage might be due to the hydroscopic nature of the rice and wheat flours, temperature, relative humidity, and nature of the packaging material (Nagi, Kaur, Dar, & Sharma, 2012;Tang et al., 2019). The difference in moisture content with packaging material agree with the study by Rao, Ramanuja, Ashok, and Vibhakar (1995) who showed that biscuits packed in metalized polyester or biaxially oriented polypropylene had higher moisture content than those packed in polyethylene bags laminated with aluminum foil. The peroxide index was neither affected by duration of storage, type of biscuit, nor packaging material. Safou biscuits recorded slightly lower peroxide index values compared to nonsafou biscuits as previously noted by Akbari, Gholami, and Ghobadi (2019). The lightness of biscuits was affected by neither storage duration nor packaging material. However, RB‐100 and RWB‐50 were lighter than the other biscuits (WB‐100, SSRWB‐50, and NSSRWB‐50). Safou biscuits were darker than nonsafou biscuits due to the brownish color of safou powder.

**Table 7:** Shelf life properties of wheat–rice biscuits affected by duration of storage, type of biscuit, and packaging material

Source	Hardness (kg)	Malondialdehyde (μmol/L)	Moisture content (%)	Peroxide index	Color (L*)
Intercept	1818.5***	0.0322***	2.54***	0.110	54.17**
Month	139.4	0.0194***	0.16***	0.392	0.39
WB−100	413.5	−0.0195**	0.42***	−0.883	2.53
RB−100	−344.7	−0.0078	−0.07	−0.819	5.68**
RWB−50	37.6	−0.0133*	−0.21*	−0.651	6.40**
SSRWB−50	350.2	−0.0155*	−0.52***	−0.428	−1.21
NSSRWB−50	0.0	0.0000	0.00	0.000	0.00
Package—aluminum	34.7	−0.0092*	−0.44***	−0.187	0.59
Package—polypropylene	0.0	0.0000	0.00	0.000	0.00
Pr > *F*(model)	**0.04**	**< 0.0001**	**< 0.0001**	**< 0.0001**	**< 0.0001**
Adjusted R^2^ (%)	6.40	55.5000	62.30	41.00	37.20

Note

Biscuit: WB‐100 = wheat biscuit; RB‐100 = rice biscuit; RSB‐100 = rice–safou biscuit; RWB‐50 = rice–wheat biscuit; SSRWB‐50 = sour safou–rice–wheat biscuit; NSSRWB‐50 = nonsour safou–rice–wheat biscuit

References: Biscuits = NSSRWB‐50; Package = Polypropylene

Ndindeng. S.A., Bella‐Manga, Kengue, J., Talle and Lewis, D.L. 2008. Quality standards for Dacryodes edulis (safou).

Research Report No. 5. International Centre for Underutilised Crops, Colombo, Sri Lanka. 26 pp.

*** *p* < .0001; **p < .001; *p < .05, L* = lightness (dark = 0; white = 100);

## 4 CONCLUSION

The fortification of rice–wheat biscuits with dry safou affected physical, sensory, and nutritional quality. RB‐100 produced with flour of fine particles (nonsandy) are harder and more cohesive than those produced with larger particles (sandy). Nonsandy biscuits recorded a higher overall acceptability score than sandy biscuits. The essential amino acids—phenylalanine, isoleucine, histidine, tyrosine, lysine—and nonessential amino acids—aspartate, glycine, alanine, gamma‐amino butyrate (GABA), proline, and arginine—contents were also higher in nonsour than sour safou. Although Leucine and aspartate were the most abundant essential and nonessential amino acids in safou, the addition of safou to rice–wheat biscuits combinations resulted in histidine increase and lysine decrease in NSRWB‐50. In addition, aspartate and glycine were increased in SSRWB‐50 and NSRWB‐50. Potassium is the most abundant mineral in safou followed by magnesium, phosphorus and calcium in that order with sodium being the least. Iron was the most abundant trace mineral in safou followed by copper, manganese, aluminum, and zinc. Potassium was still the most abundant mineral in rice biscuits produced followed by phosphorus. Although the trace minerals iron and zinc were higher in rice biscuits than in safou, there was higher quantity of aluminum in those biscuits than in safou but this quantity is comparable with data in literature. Thus, the biscuits produced can be a good source for potassium, phosphorus, iron, and zinc for different age and sex groups. Highly acceptable biscuits could be obtained when 30% of sour or nonsour safou powder was used in the formulation. The small variation of peroxide and MDA values in fortified biscuit indicated slight oxidative activity that changed little during storage irrespective of the packaging material used. The contribution of safou powder in making biscuits enabled an increase in protein and mineral content that would contribute in the fight against malnutrition within vulnerable groups of the population. The use of broken rice and safou could reduce postharvest losses recorded in these crops and increase the income of those involved in this sector especially women processors.

## CONFLICT OF INTEREST

The authors declare that they do not have any conflict of interest.

## ETHICAL REVIEW

“This study was approved by the Institutional Review Board of Yaounde‐1 University.”

## INFORMED CONSENT

Written informed consent was obtained from all study participants.

## Supporting information

Supplementary Material
