# Supplementary material for: Physical, nutritional, and sensory quality of rice‐based biscuits fortified with safou (Dacryodes edulis) fruit powder
Source: Food Sci Nutr. 2020 May 16;8(7):3413–24. doi: 10.1002/fsn3.1622 (PMC7382197; doi:10.1002/fsn3.1622)
Supplement: Supplementary file 1 — Supplementary Material [file FSN3-8-3413-s001.docx]

Optimization of wheat, rice, butter and safou for rice-wheat-safou biscuits production

# Methodology

Twenty-one wheat, rice, butter and safou biscuit combination with two replications were generated using the Doehlert design in XLSTAT Premium version 2020.1.2 (Addinsoft, 2020). The different biscuits were produced and submitted to a trained panel of 8 persons for sensory evaluation. The parameters that were appreciated were particle size, melty, shape, crispiness, hardness, aroma and overall quality. The data was analyzed in MINITAB statistical software verion 14.13 using the Response Surface design analysis. All analyses were done at the 5% significance level.

# Results and discussions.

Below are the equations for the different parameters evaluated and how each parameter is affected by wheat (X_1_), rice (X_2_), butter (X_3_), safou (X_4_) or their interactions.

## Particle size

Particle size = 1.83 + X_1_0.00026 + X_2_0.00108 + X_3_0.00104 + X_4_0.00746 + Ƹ; (R^2^=11.9, Lack-of-Fit = P<0.0001) Eqn 1

Rice flour had a positive effect on particle size (P<0.000), however, the addition of safou to rice biscuits reduces particle size (P<0.009)

## Melty

Melty = 2.38 - X_1_0.00076 - X_2_0.00235 + X_3_0.00536 - X_4_0.00222 + Ƹ; (R^2^=23.6, Lack-of-Fit = P<0.013) Eqn 3

Rice flour negatively influenced the ability of biscuits to melt while chewing (P < 0.043). Rice-wheat combination had a positive effect on melty (P<0.0000) as well as wheat-safou (butter (P< 0.006).

## Shape

Shape = 4.17 + X_1_0.00162 - X_2_0.00036 - X_3_0.00305 - X_4_0.00241 + Ƹ; (R^2^=24.4, Lack-of-Fit = P<0.0001) Eqn 4

Wheat flour positively influences the shape of biscuit (P< 0.039) as well as rice flour and butter interaction (P<0.003)

## Crispiness

Crispiness = 2.97 + X_1_0.00037 + X_2_ 0.00416 + X_3_0.00019 - X_4_0.00145 + Ƹ; (R^2^=50.3, Lack-of-Fit = P.> 0.05) Eqn 4

Rice flour positively influences crispiness of biscuits (P<0.001) while the addition of safou to rice flour decreases crispiness (P=0.029). Generally, the addition of butter and safou negatively influence crispiness of biscuits (P<0.012)

## Hardness

Hardness = 3.62 + X_1_0.00197 + X_2_0.00213 - X_3_0.00898 - X_4_0.00183 + X_3_^2^0.00001 + Ƹ; (R^2^=50.3, Lack-of-Fit = P > 0.05) Eqn 5

Wheat flour had a positive effect of the hardness of biscuits (P<0.014). Rice-safou interaction had a negative effect on hardness (P<0.029) while safou and butter generally decreases hardness of biscuits (P<0.012)

## Aroma

Aroma =3.30 - X_1_0.00060 - X_2_0.00024 + X_3_0.00296 - X_4_0.00020 + Ƹ; (R^2^=21.1%, Lack-of-Fit = P > 0.05) Eqn 6

None of the four ingredients; wheat, rice, butter and safou influenced the aroma of the biscuit.

## Overall quality

Overall quality = 4.98 - X_1_0.00024 - X_2_0.00171 + X_3_0.00027 - X_4_0.00811 + X_4_^2^0.00001 + Ƹ; (R^2^=49%, Lack-of-Fit = P < 0.05) Eqn 6

Wheat and butter interaction and safou respectively influenced negatively the global quality (P=0.029) and (P<0.0001)

## Response Optimization

The response optimization is shown in Table 1 and 2 below.

Table 1. Starting point and local solution to produce wheat-rice-safou biscuit combinations.

| Ingredient | Starting point | Local solution |
| --- | --- | --- |
| Wheat (g) | 600 | 496 |
| Rice (g) | 600 | 601 |
| Butter (g) | 400 | 468 |
| Safou (g) | 120 | 62 |

Table 2. Predicted responses and desirability when local solution is used.

| Parameters | Predicted response | Desirability |
| --- | --- | --- |
| Particle size | 3.5 | 0.51 |
| Melty | 2.6 | 0.23 |
| Shape | 3.2 | 0.42 |
| crispiness | 3.9 | 0.66 |
| Hardness | 2.7 | 0.23 |
| Aroma | 3.4 | 0.47 |
| Overall quality | 4.0 | 0.67 |

# Conclusion

Hardness and crispiness equations can be used for predicting these parameters for wheat-rice-safou biscuits combinations. Particle size and crispiness are important parameters to watch when producing rice-wheat-safou biscuits. It is possible to produce wheat-rice biscuits with the partial substitution of butter with safou which are highly acceptable (4.0 on a scale of 5).

# Reference

Addinsoft (2020). XLSTAT statistical and data analysis solution. Boston, USA. <https://www.xlstat.com>.
